# Function and Regulation of microRNA171 in Plant Stem Cell Homeostasis and Developmental Programing

**DOI:** 10.3390/ijms23052544

**Published:** 2022-02-25

**Authors:** Han Han, Yun Zhou

**Affiliations:** 1 Department of Botany and Plant Pathology, Purdue University, West Lafayette, IN 47907, USA; hanhan.bio@gmail.com; 2 Purdue Center for Plant Biology, Purdue University, West Lafayette, IN 47907, USA

**Keywords:** microRNA, mobile signal, plant development, stem cells, meristems, evolution

## Abstract

MicroRNA171 (miR171), a group of 21-nucleotide single-strand small RNAs, is one ancient and conserved microRNA family in land plants. This review focuses on the recent progress in understanding the role of miR171 in plant stem cell homeostasis and developmental patterning, and the regulation of miR171 by developmental cues and environmental signals. Specifically, miR171 regulates shoot meristem activity and phase transition through repressing the *HAIRY* *MERISTEM* (*HAM*) family genes. In the model species Arabidopsis, miR171 serves as a short-range mobile signal, which initiates in the epidermal layer of shoot meristems and moves downwards within a limited distance, to pattern the apical-basal polarity of gene expression and drive stem cell dynamics. miR171 levels are regulated by light and various abiotic stresses, suggesting miR171 may serve as a linkage between environmental factors and cell fate decisions. Furthermore, miR171 family members also demonstrate both conserved and lineage-specific functions in land plants, which are summarized and discussed here.

## 1. Introduction

MicroRNAs (miRNAs) are 20–24 ribonucleotide single-strand non-coding small RNA molecules, which regulate gene expression post-transcriptionally [1,2]. In animals, plants and fungi, miRNAs recognize their target messenger RNAs (mRNAs) through complementary base pairing, leading to the repression of gene expression at the transcriptional and post-transcriptional level [1,2,3]. The firstly identified endogenous miRNAs are *lin-4* and *let-7*, which control the timing of larval development in the nematode *Caenorhabditis elegans* [4,5]. In plants, a considerable number of studies have demonstrated that miRNAs play vital roles in various processes, including developmental programming and responses to both biotic and abiotic stresses [1,2,3,6,7]. miRNAs share features with the small interfering RNAs (siRNAs) of the RNA interference (RNAi pathway) [8]; however, one of the differences between these two types of small RNAs is that miRNAs are endogenous products, while siRNAs are primarily exogenous in their origin, such as the virus and transgenes [8].

Plant miRNAs not only serve as local signals, but also can function non-cell-autonomously [9]. Mobile miRNAs travel from one cell to another, likely via plasmodesmata in plants [10,11,12]. Several miRNA species move over a long distance within a plant through vascular tissues or even between the plants and their interacting organisms, such as parasitic plants and pathogens [12,13,14,15,16,17]. Recent studies demonstrated that several miRNA species travel via the environment from one plant to its neighboring plant and induce gene silencing [17,18]. These results suggest that exogenous miRNAs, potentially as signaling molecules, are able to post-transcriptionally regulate expression of endogenous genes in plants [17,18]. In the model species of flowering plants, Arabidopsis, the cell-to-cell movement of miRNAs relays key positional signals during plant morphogenesis [19,20,21,22,23,24,25,26,27]. For example, the concentration gradient of the miR165/166 family members specifies the adaxial–abaxial polarity in developing leaves and determines the central stele fate in roots [20,22,23,28,29]; a recent study demonstrated that two related receptor-like kinases, *BARELY ANY MERISTEM* (*BAM*) *1* and *BAM2*, are required for the movement of miR165/166 in roots [29]. In Arabidopsis shoot apical meristems (SAMs), the movement of miR394 from the epidermis to inner cell layers is essential to maintain stem cell competency [24,30]. In addition, the cell-to-cell movement of miRNAs seems to be directional, suggesting a yet-undefined mechanism that gates their movement [31]. In this review, we summarize the recent progress in understanding the function and regulation of microRNA170/171 (hereafter referred to as miR171), a group of conserved miRNA species widely present in land plants. We discuss how they act as a short-range mobile signal in patterning gene expression in shoot meristems and how they are regulated in response to environmental signals and developmental cues.

## 2. miRNA171 and Its Targets

**Figure 1:**
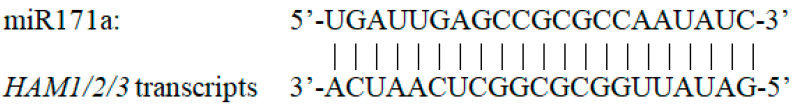
The sequence of miR171a is complementary to the transcripts of Arabidopsis *HAM1 HAM2* and *HAM3* genes, which belong to the Type II group in the HAM family.

In general, plant *MIRNA* genes are transcribed into relatively long primary transcripts (pri-miRNAs), which are subsequently processed into shorter pre-miRNAs [32]. pre-miRNAs fold into characteristic stem-loop hairpin structures, which are cleaved by DICER-LIKE (DCL) proteins, forming 21- to 24-nt miRNA/miRNA* duplexes. The antisense miRNA* is then degraded and the mature miRNA is embedded into an RNA-induced silencing complex (RISC) that contains the ARGONAUTE (AGO) proteins [33,34,35]. miR171 represents a small group of the 21-nuleotide single-strand micro RNAs [1,6,7,36,37]. In Arabidopsis, the total miR171 species are from the products of four genes located at different regions of the genome, including *MICRORNA171A* (*MIR171A*), *MICRORNA171B* (*MIR171B*), *MICRORNA171C* (*MIR171C*) and *MICRORNA170* (*MIR170*). The *MIR170* and *MIR171A* genes were initially identified in 2002 [1,6,36,37] and the *MIR171B* and *MIR171C* genes and their products were identified two years later [7]. The sequence of mature miR171a (UGAUUGAGCCGCGCCAAUAUC) is perfectly complementary to its target mRNAs (Figure 1). In contrast, miR171b and miR171c have the identical miRNA sequences (UUGAGCCGUGCCAAUAUCACG) [7], which are slightly different with that of miR171a. In addition, the mature miR170 (UGAUUGAGCCGUGUCAAUAUC) differs from miR171a at the 12th and 14th positions, with two base pair mismatches to their targets [36]. Arabidopsis miR171 specifically targets and represses three *HAIRY MERISTEM* (*HAM*) family genes [1,6,7,36,37], which are also named as *LOST MERISTEM* (*LOM*) and *SCARECROW-LIKE* (*SCL*) genes in different plant species [6,38,39,40]. *HAM* family genes encode the GRAS (GAI, RGA and SCR) domain transcription factors [41,42,43], which belong to the GRAS superfamily [41,44]. There are four *HAM* family genes (*HAM1-4*) in Arabidopsis [41,45]. Among them, *HAM1*, *HAM2* and *HAM3* contain the conserved miR171-binding sequence, 5′-GATATTGGCGCGGCTCAATCA-3′, within their coding sequences [41,45] and their transcripts are specifically targeted by miR171 (Figure 1) [36,37,38]. In contrast, Arabidopsis *HAM4* (*AtHAM4*) is classified into a different group and *HAM4* lacks a conserved miR171 target site [41,45]. *HAM* family genes regulate multiple growth and developmental processes in Arabidopsis [38,39,40,41,42,43,45,46,47,48,49], including the control of stem cell identity and homeostasis in shoot apical meristems and axillary meristems. Shoot meristems harbor a small group of undifferentiated stem cells, maintaining a balance between the self-renewal of stem cells and cell differentiation into lateral organs. In shoot meristems, the miR171-regulated *HAM1* and *HAM2* play crucial and shared function in control of the *WUSCHEL* (*WUS*)-*CLAVATA3* (*CLV3*) feedback loop and drive the stem cell homeostasis [41,42,45,46,47,48,49,50,51]. A recent work demonstrated that *HAM3*, the other *HAM* member regulated by miR171 in Arabidopsis, plays a dispensable or minor role in determining the apical-basal patterning of shoot meristems, but likely shares redundant functions with *HAM1* and *HAM2* in control of other aspects of shoot and root development [42,48].

Several miR171 species also target the *NSP2* (*NODULATION SIGNALING PATHWAY 2*) family members in *Medicago truncatula*. As an example, Medicago miR171h (Mtr-miR171h, 5′-CGAGCCGAAUCAAUAUCACUC-3′) specifically recognizes and cleaves the transcript of *MtNSP2. MtNSP2* also encodes a GRAS domain protein, which functions as a transcriptional activator and plays a key role in both arbuscular mycorrhizal colonization and nodulation signaling pathways [52,53,54,55]. The *NSP2* family is sister to the *HAM* family, and they both belong to the GRAS superfamily. Although *NSP2* orthologs are widely distributed in land plants and the cleavage of *NSP2* family members by miR171 is also found in other plant species, including *Lotus japonicus* [56,57,58], a phylogenetic study suggests that the miR171h-NSP2 regulatory module is only conserved in the angiosperms that enable arbuscular mycorrhizal symbiosis [53].

The *SU(VAR)3-9 HOMOLOG8* (*SUVH8*)/*SET DOMAIN GROUP 21* gene is regulated by the *MIR171/170* products through a different mechanism [59]. During microRNA biogenesis, usually the guide strand (named as miRNA) is retained and the passenger strand (named as miRNA*) is degraded, subsequent to association of the miRNA/miRNA* duplex with RISCs [32]. Under certain circumstances, the miRNA* is retained as the guide strand [60]. In Arabidopsis, miR170* and miR171a* trigger the tissue-specific silencing of *SUVH8* [59,61]. miR171a* was preferentially loaded into AGO1 and *SUVH8* mRNA degradation products were identified corresponding to the miR171a*-mediated cleavage [59]. The plants that specifically altered the miR171a*-*SUVH8* balance (through overexpressing miR171a* target mimic or expressing the miR171* resistant *SUVH8*) showed dwarfed stature, long and curved leaves and reduced fertility [59]. The miR171a* target site in *SUVH8* is only found in the Arabidopsis lineage, seems not to be conserved across species [59]. The molecular mechanism underlying the selection and regulation of miR171*/miR170* as the guide strand in Arabidopsis under different physiological and environmental contexts deserves further studies [62,63].

## 3. Function of miR171

**Figure 2:**
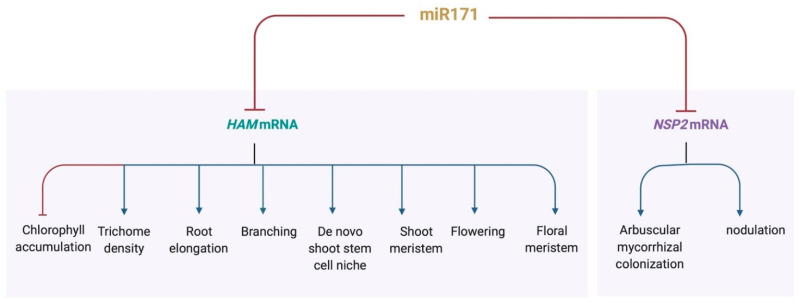
miR171 functions as a regulatory hub that participates in diverse developmental processes. Created with BioRender.com.

In Arabidopsis, miR171 plays an essential role during shoot meristem development. The overexpression of miR171 in Arabidopsis results in the defects in shoot stem cell initiation and maintenance, with the mis-regulated *CLV3* expression pattern, disorganized meristem structure, and reduced shoot branches [38,42,47,48,49], which are comparable to the phenotypes of the *ham1ham2ham3* triple or *ham1ham2* double loss of function mutants [38,41,42,46,47,48]. A 3D computational model predicts that the partial loss of function of *HAM* genes induced by miR171 overproduction is sufficient to disturb the apical-basal pattern of the *CLV3* expression domain in Arabidopsis SAMs [47]. This simulation has been validated by RNA in situ hybridization assays in the *35S::MIR171* plants, showing the *CLV3* mRNA pattern with the greatly reduced (but detectable) expression in the first two upper layers and the increased level in the deep cell layers of the SAMs [47]. Furthermore, in *35S::MIR171* transgenic plants, the *CLV3* expression in developing axillary meristems is always confined into deep cell layers, demonstrating the role of miR171 in both established and de novo stem cell niches [48]. Besides the shoot meristem defects, the miR171 overexpression plants also display the altered shoot architecture, leaf shape, leaf color and chlorophyll accumulation, flower structure, trichome morphology and primary root elongation, suggesting that miR171 functions a regulatory hub in control of diverse developmental processes (Figure 2) [38,39,40,41]. Among them, the miR171-HAM pathway regulates phase transition and trichome distribution, likely through inhibiting the activity of miR156-targeted *SQUAMOSA PROMOTER BINDING PROTEIN LIKE* (*SPL*) genes [39], the conserved and key regulator of phase transition [64,65,66]. In addition, the key role of miR171 in plant development is revealed by the silent mutations in the miR171 binding sites of the *HAM* genes, which lead to the miR171 uncleavable (insensitive) targets. These mutated *HAM* genes produces mRNAs that cannot be recognized by miR171 but in which the amino acid sequences of encoded proteins are unchanged [38,67]. When the miR171 insensitive HAM1 is specifically expressed in the epidermal layer, it leads to the reduced *CLV3* expression in the epidermis and abnormal meristem development [46]. The accumulation of the miR171 insensitive *HAM* genes also results in developmental defects in cotyledons, young seedlings, leaves and shoot branching [38,67]. All these results suggest that it is crucial to spatially confine the HAM expression domain by miR171 in Arabidopsis.

The role of miR171 in regulating meristem identity seems to be highly conserved across flowering plants. As a result, the miR171-HAM regulatory module controls important agronomic traits in crops. For example, overexpression of Sly-miR171 in tomato leads to disturbed shoot development [56,68,69], which is similar to phenotypes of the *ham* loss-of-function mutants in petunia [43] and in pepper [70]. Utilizing a two component system, overexpression of either Sly-MIR171a or Sly-MIR171b leads to enlarged meristems and growth arrest after producing few leaves in tomato [56]. In monocots, the overexpression of osa-miR171c in rice (*Oryza sativa*) leads to the enlarged shoot apex and the misregulation of key meristem regulators, including the rice orthologs of *CLV1*, *CLV3* and *WOX4* [71]. The overexpression of miR171 in barley (*Hordeum vulgare*) results in similar phenotypes, including the reduced shoot branching [72]. In addition, miR171 participates in developmental phase transition in monocots (including barley and rice), likely through the conserved miR156-SPL signaling circuit [71,72].

miR171h negatively regulates arbuscular mycorrhizal and nodule symbiosis by targeting *MtNSP2* in Medicago [52,53,54,73] (Figure 2). Fungal colonization and nodulation numbers was reduced in the roots of miR171h overexpression plants, similar to *nsp2* loss of function mutants [53,74,75]. In addition, the miR171h-NSP2 module regulates root endosymbiosis likely through the phytohormone cytokinin responsive signaling [54]. Interestingly, Medicago miR171b (Mtr-miR171b) has evolved a target site mismatch at the identified cleavage site, which protects its target gene *MtHAM1* from the cleavage by other miR171 family members. As a result, arbuscular mycorrhizal symbiosis is stimulated [73]. This regulation involving Mtr-miR171b seems to be specific in mycotrophic species [73]. In rice roots, a tissue-specific transposon transcribes competing endogenous RNAs, which also act as target mimics and interrupt the function of miR171, protecting the targets—*OsHAM* genes from the degradation [76]. In the future, it will be interesting to explore whether similar mechanisms exist in other miRNA families and in other biological processes.

The function of miR171 in flowering plants also has been uncovered by expressing the miRNA target MIMICs (MIMs) or short tandem target MIMICs (STTMs), which are uncleavable targets for sequestering miRNAs into non-productive interactions [77,78]. The *35S:MIM171a* and *35S:MIM170* transgenic Arabidopsis plants showed similar phenotypes, with round leaves in pale green, defective anthesis and reduced fertility [79]. On the contrary, the *35S:MIM171b* and *35S:MIM171c* plants are comparable to the wild type control, which is likely due to the genetic redundancy and partial subfunctionalization among members of the *MIR171/170* family. The STTM transgenic line silencing miR171 also lead to the developmental defects in the monocot rice, including the semi-dwarf stature, enclosed panicles, and drooping flag leaves [80]. Overexpression of STTM171 in tomato results in irregular compound leaf morphology, increased branch number and male sterility, demonstrating the role of sly-miR171 in regulating axillary meristem activity, compound leaf development and anther development [81].

## 4. Expression Patterns and Regulation of miR171

The miR171 precursors and mature miR171 species are widely accumulated in different tissues of Arabidopsis, including cotyledons, hypocotyls, leaves, flowers and roots [1,36,38,59,82]. Confocal imaging of the fluorescent transcriptional reporters demonstrated that the *MIR171A* gene is highly and specifically expressed in the epidermal layer of embryos, vegetative SAMs, young leaves and stems, inflorescence SAMs and floral meristems [67,82,83]. In addition, the promoter activities of the other three *MIR170/171* family genes (*MIR171B*, *MIR171C* and *MIR170*) are also specifically turned on in the epidermis of shoot meristems at both vegetative and reproductive stages, though the expression levels of the *MIR171C* and *MIR170* genes are weaker than that of *MIR171A* and *MIR171B* [82]. These findings suggest that the epidermis is the major synthesis site for miR171 in Arabidopsis shoots. On the contrary, these *MIR171* genes are not expressed in the root epidermis [82], suggesting *MIR171* expression patterns in shoots and roots are controlled by different mechanisms.

**Figure 3:**
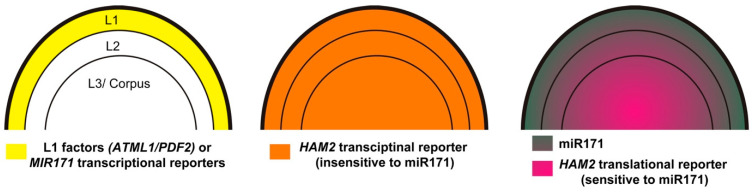
Diagrams illustrating the expression patterns of the L1-specific transcription factors (ATML1 and PDF2) and *MIR171* transcriptional reporters (**left**), the miR171-insensitive *HAM2* transcriptional reporter (**middle**), and the mature miR171 and the miR171-sensitive *HAM2* translational reporter (**right**) in Arabidopsis SAMs. The Arabidopsis SAM consists of three clonally distinct cell layers, including the L1, L2, and L3/corpus, which are indicated here. L1 represents the epidermal layer, L2 represents the subepidermal layer, and L3/corpus includes all the cells beneath the subepidermal layer.

The miRNA in situ hybridization has shown that the localization of mature miR171 is not only highly accumulated in the epidermal layer but also detectable in the second and third cell layers of the SAM [82]. In addition, the comparison between the expression patterns of the miR171-sensitive and miR171-insensitive *HAM* reporters in the SAMs serves as a direct readout of the miR171 localization (Figure 3) [82]. For example, a miR171-insensitive *HAM2* transcriptional fluorescent reporter is ubiquitously expressed in all the layers of Arabidopsis SAMs [82]. In contrast, the miR171-sensitive *HAM2* translational fluorescent reporter is highly expressed in deep cell layers but repressed in the upper layers, forming a concentration gradient along the apical-basal axis of SAMs (Figure 3). This concentration gradient is complementary to the pattern of mature miR171 in the SAMs [82] and crucial for the apical-basal patterning of SAMs [47] (Figure 3). All these results suggest that miR171 is a short-range mobile signal, moving downwards across one to two cell layers and shaping the apical-basal patterns of HAM proteins in Arabidopsis SAMs [67,82]. The highly restricted movement of miR171 within Arabidopsis SAMs is consistent with the findings that the cell-to-cell movement of microRNAs in general is highly controlled and limited in Arabidopsis SAMs [31,82]. It will be interesting to explore how the movement of miR171 is governed in the future studies. In addition, although the function of miR171 family in control of root endosymbiosis is well studied as described above, whether miR171 moves within roots and whether miR171 serves as a short-range mobile signal in this process needs further studies.

The epidermis-specific expression of Arabidopsis *MIR171* genes is directly determined by two closely related homeodomain leucine zipper class IV (HD-ZIP IV) transcription factors—ARABIDOPSIS MERISTEM LAYER 1 (ATML1) and PROTODERMAL FACTOR 2 (PDF2) [82]. ATML1 and PDF2 function as key regulators in the epidermal specification pathway and they both are specifically expressed in the epidermal layer of SAMs and floral meristems [84,85,86,87,88]. Both the yeast one hybrid and gel mobility shift assays demonstrated that ATML1 and PDF2 proteins directly bind to the promoters of *MIR171* genes. The conserved L1-box motifs in the promoters of *MIR171* genes, which serve as consensus binding sites for ATML1/PDF2, mediate the physical interactions between the *MIR171* promoter DNAs and ATML1/PDF2 proteins. These L1-box motifs are also required for the activation of *MIR171* genes by ATML1, because *MIR171* genes fail to express in the L1 layer once these cis-elements are mutated [82]. In addition, compared to that in wild type, expression of the *MIR171* reporters is absent or greatly reduced in the *atml1 pdf2* double mutants, demonstrating that ATML1/PDF2 are required for the *MIR171* expression in Arabidopsis (Figure 3) [82]. Using ATML1/PDF2 as a functional input, a 3D computational model was established to simulate and quantitatively evaluate the function of a L1(ATML1/PDF2)-miR171-HAM signaling cascade in Arabidopsis SAMs. Both in silico prediction and in vivo experimental results demonstrated that the transient activation of ATML1 in the SAMs rapidly induce the *MIR171* promoter activities, subsequently leading to the reduction of *HAM* expression [82], suggesting an essential role of the L1-miR171-HAM signaling cascade in Arabidopsis SAMs.

The expression of miR171 is regulated by multiple environmental signals [7,89,90]. Determined by the RNA gel blot, the miR171 level increases during the light period of the daytime and decreases in the dark period, and this oscillatory pattern of miR171 accumulation is governed by light instead of the circadian clock [89]. The light-induced miR171 expression might have a connection with the findings that the miR171-HAM pathway affects chlorophyll biosynthesis under light [40]. Interestingly, osa-miR171 species in rice are also induced by light [71]. Their accumulation peak in the early morning and the expression of four rice *HAM* homologs show the complementary fluctuations [71]. Besides light, miR171 responds to stresses in different plant species [90,91,92,93]. Through comprehensive microarray expression analyses in Arabidopsis, it was found that several miR171 species (miR171b and miR171c) are induced by abiotic stresses, including high salinity, drought and low temperature [90]. Small RNA profiles generated by next-generation sequencing in potato demonstrated that the potato miR171a, miR171b and miR171c are also drought-induced [91]. In addition, Mtr-miR171h expression is induced in mycorrhizal roots and root nodules in Medicago [52,75]. It is also induced by high phosphate nutrition and repressed by phosphate starvation [52,75], suggesting a potential linkage between the nutritional status and endosymbiosis of plants [52,53,54,55]. All these findings lead to a hypothesis that miR171 likely serves as a signaling bridge linking environmental factors to plant growth and development. miR171 also demonstrates high expression in undifferentiated callus from a number of species, including rice, Japanese larch (*Larix kaempferi*), orange (*Citrus sinensis L. Osb.*) and lilies (*Lilium pumilum*) [94,95,96,97,98,99,100]. It will be interesting to determine whether miR171 involves callus formation, regeneration or somatic embryogenesis in the future.

The miR171 abundance is also positively regulated by a small peptide generated from its own pre-miRNA, suggesting an additional layer of regulation of miR171 [101,102]. In Arabidopsis, Medicago and grape, the primary transcripts of miRNAs encode small regulatory peptides called the miRNA-encoded peptides (miPEPs), which specifically activate the transcription of their own miRNAs [101,102]. An exogenous miPEP also sufficiently activates the production of its related miRNA in plants, suggesting potential agronomical applications. This regulation seems to be conserved in several different miRNA families. As an example, the Medicago miR171b produces miPEP171b, which in turn enhances the production of miR171b and plays a role in arbuscular mycorrhizal symbiosis [73]. It will be interesting to explore the molecular basis by which miPEPs positively regulate miRNAs.

## 5. Evolution of miR171 in Land Plants

The miR171 family is one of the ancient *MIRNA* families that have been identified in the common ancestor of all embryophytes [103]. miR171 is broadly present across land plants, from bryophytes to angiosperms (flowering plants) [103,104]. Consistently, their targets, the *HAM* family genes, are also widely present in land plants and they likely originated prior to the divergence of bryophytes [41,45]. The miR171 binding site, 5′-GATATTGGCGCGGCTCAATCA- 3′, seems to be an ancestral trait for the *HAM* family [45]. This site is highly conserved in almost all the non-angiosperm *HAM* members examined to date, including the *HAM* homologs identified in bryophytes, lycophytes, ferns and gymnosperms [41,45,105]. In angiosperms, the *HAM* family expanded and diversified into two distinct groups: Type I and Type II [41,45,106]. The miR171-binding sequence is retained in most Type II *HAM* members but lost in the majority of Type I *HAM* members [41,45]. Confocal imaging results demonstrated that the conserved miR171-binding sites in the *HAM* homologs from different species are recognized by miR171 in Arabidopsis SAMs, leading to the cleavage similar to endogenous Arabidopsis *HAM2* transcripts [45]. Thus, the conserved miR171-binding sequence in these species suggests strong selective pressure in retaining this regulation and limiting *HAM* expression. Furthermore, the specific repression of *HAM* expression by miR171 is likely crucial for the conserved functions of the *HAM* family [45], which needs future studies. Recently, it was found that in the moss *P. patens*, the miR171-regulated *PpHAM*/*PpGRAS12* plays a role in meristem formation [107] and the *PpHAM/PpGRAS12* overexpression induces the enlarged and multiple apical meristems. The role of miR171 is also suggested in the liverwort *Marchantia polymorpha*, based on the RNA-seq and degradome analyses [108,109].

The number of *MIR170/171* family genes demonstrated dramatic variations among different species (as per miRBase) [110]. The miR171 precursors also display high diversifications and members of the *MIR170/171* family likely evolved in a lineage-specific manner [110,111,112,113]. In addition, the promoters of *MIR170/171* family genes have undergone most sequence diversification within species, suggesting that different members may have evolved specialized expression patterns to differentially regulate or fine tune target genes [110]. In contrast, the mature miR171 sequences are highly conserved in a number of flowering plants. In four plant species, including Arabidopsis, poplar (*P. trichocarpa*), rice and grape (*V. vinifera*), 21 out of 34 miR171 sequences are nearly identical, with only different nucleotides at one or two positions [110]. In addition, a separate study of the miR171 phylogeny in several fruit species leads to a similar conclusion [113]. Nevertheless, despite strong conservation of mature miR171 sequences in general, a small proportion of miR171 species have critical sequence variations and target non-*HAM* genes as described above [52,56,110,114].

## 6. Future Directions and Perspectives

In Arabidopsis shoot apical meristems, miR171 only moves across one or two cell layers, leading to the question whether the mobility of miRNA is a characteristic of the specific miRNA or of the specific cell type. It will be interesting to explore whether the cell-to-cell movement of miR171 is also highly restricted in leaves, roots and stems, and whether its movement in these tissues involves key developmental decision and cellular patterning as well. The level of miR171 is directly controlled by the epidermal specification pathway (the ATML1 and PDF2 transcription factors) [82], and it is also induced by environmental signals, such as light and abiotic stresses [7,89,90]. It will be very informative to determine molecular mechanisms by which miR171 links environmental factors to plant development. Furthermore, miR171 is widely present in land plants, however, its function and regulation in non-seed plants is largely unknown. The future studies of miR171 in seed-free vascular plants, such as in the fern *Ceratopteris richardii* [44,115,116], will provide more insights into the evolution of this key miRNA family in land plants.

## Data Availability

Not applicable.
